# Flying with the wind: scale dependency of speed and direction measurements in modelling wind support in avian flight

**DOI:** 10.1186/2051-3933-1-4

**Published:** 2013-07-03

**Authors:** Kamran Safi, Bart Kranstauber, Rolf Weinzierl, Larry Griffin, Eileen C Rees, David Cabot, Sebastian Cruz, Carolina Proaño, John Y Takekawa, Scott H Newman, Jonas Waldenström, Daniel Bengtsson, Roland Kays, Martin Wikelski, Gil Bohrer

**Affiliations:** Department for Migration and Immuno-ecology, Max Plank Institute for Ornithology, Am Obstberg 1, 78315 Radolfzell, Germany; Department of Biology, University of Konstanz, Konstanz, 78464 Germany; Am Fügsee 29, Seehausen am Staffelsee, 82418 Germany; Wildfowl & Wetlands Trust, Slimbridge, Gloucestershire, GL2 7BT UK; Environmental Consultancy Services, White Strand, Killadoon, Louisburgh, Westport, Co. Mayo Ireland; U.S. Geological Survey, Western Ecological Research Center, 505 Azuar Drive, Vallejo, CA 94592 USA; Emergency Center for Transboundary Animal Diseases, Animal Production and Health Division, Food & Agriculture Organization of the United Nations, Rome, 00153 Italy; Centre for Ecology and Evolution in Microbial Model Systems (EEMiS), Linnaeus University, Kalmar, SE-391 82 Sweden; School of Natural Resources, North Carolina State University, 3118 Jordan Hall, Raleigh, NC 27695 USA; North Carolina Museum of Natural Sciences, 11 West Jones St, Raleigh, NC 27601 USA; Department of Civil, Environmental & Geodetic Engineering, The Ohio State University, Columbus, OH 43210 USA

**Keywords:** NOAA, ECMWF, GPS, Aves, Doppler-shift, Scaling, Measurement error, Flight direction, Flight speed

## Abstract

**Background:**

Understanding how environmental conditions, especially wind, influence birds' flight speeds is a prerequisite for understanding many important aspects of bird flight, including optimal migration strategies, navigation, and compensation for wind drift. Recent developments in tracking technology and the increased availability of data on large-scale weather patterns have made it possible to use path annotation to link the location of animals to environmental conditions such as wind speed and direction. However, there are various measures available for describing not only wind conditions but also the bird's flight direction and ground speed, and it is unclear which is best for determining the amount of wind support (the length of the wind vector in a bird’s flight direction) and the influence of cross-winds (the length of the wind vector perpendicular to a bird’s direction) throughout a bird's journey.

**Results:**

We compared relationships between cross-wind, wind support and bird movements, using path annotation derived from two different global weather reanalysis datasets and three different measures of direction and speed calculation for 288 individuals of nine bird species. Wind was a strong predictor of bird ground speed, explaining 10-66% of the variance, depending on species. Models using data from different weather sources gave qualitatively similar results; however, determining flight direction and speed from successive locations, even at short (15 min intervals), was inferior to using instantaneous GPS-based measures of speed and direction. Use of successive location data significantly underestimated the birds' ground and airspeed, and also resulted in mistaken associations between cross-winds, wind support, and their interactive effects, in relation to the birds' onward flight.

**Conclusions:**

Wind has strong effects on bird flight, and combining GPS technology with path annotation of weather variables allows us to quantify these effects for understanding flight behaviour. The potentially strong influence of scaling effects must be considered and implemented in developing sampling regimes and data analysis.

**Electronic supplementary material:**

The online version of this article (doi:10.1186/2051-3933-1-4) contains supplementary material, which is available to authorized users.

## Background

Bird flight has long fascinated humanity and much research has been devoted to understanding the mechanics, evolution, and limitations of bird flight [1–3]. Central to many of these questions is the understanding of how atmospheric conditions, mainly wind, influence local movements and migration strategies [4–9]. Laboratory-based approaches using wind tunnels have provided insight into the physiological, biomechanical and morphological adaptations which enable birds to fly [10–13]. Beyond these laboratory experiments, however, there remains the challenge of determining factors important for optimizing the movement of wild animals in their natural environments which underlie the ecological and evolutionary processes that have shaped current patterns of bird flight and migratory behavior [14–16].

Understanding behavioral or physiological responses to environmental conditions is a central question in biology. Recent technological advances have made it possible to use tracking technology to record the flight path of wild birds and then determine from weather records the atmospheric conditions at the locations where the birds were recorded [17–20]. This approach, known as path annotation, estimates key metrics needed for understanding the effect of wind on birds under natural conditions including the speed of the animal relative to the ground (ground speed) and air (airspeed). By accounting for the recorded direction of the movements, parameters relating to orientation, navigation, and compensation for wind drift can be estimated [21–25]. These measures can then be used to address important ecological and evolutionary questions such as costs of migration (in terms of travel time, flight effort and allocation of fat reserves), as well as movement decisions (*e*.*g*. stopping to refuel energy reserves or continuing to fly) [10, 26, 27]. Ground speed, and thus flight efficiency, are key determinants in the theory of optimal migration in the field [8, 13, 28]. The difficulty of collecting data at an appropriate scale has hitherto resulted in a limited quantification of flight speed of birds, especially for migration over many thousands of kilometers through constantly changing weather conditions.

The development of miniaturized satellite tracking devices in the 1990s made it possible to study birds moving at continental to global scales [29]. However, the poor spatial accuracy of the early location data (typically in the range of 1-100 km) limited the use of those studies for key questions requiring higher spatio-temporal resolution such as the influence of wind on direction and speed of migration [30]. The advent of GPS tracking devices, and further miniaturization of transmitters and loggers, has recently made it practical to study animal movement in previously unachieved detail and precision. Tags now are capable of recording locations several times per second with a spatial accuracy of 3m and less [31]. At the same time, an easement of government policies on the use and sharing rights of climate data has facilitated access to global scale, hybrid model-observation weather-reanalysis datasets. Modern web-based tools have made it easier to store and visualize movement data [32] and weather models, and to link recorded locations of an animal with concurrent environmental conditions [17, 19, 33]. Thus, both the flight and environmental data are now available to address questions on how free-ranging birds migrate over large distances through dynamic weather systems.

Here, we compare measured ground speed (*v*_*g*_) and estimated as well as calculated airspeed (*v*_*a*_) for 288 birds from nine different species tracked with GPS tags in relation to the different methods used for calculating flight direction (*d*) and ground speed (*v*_*g*_), and two sources of weather data. By doing so, we provide the basic data needed to determine the relationship between the animals' speeds and the wind conditions encountered during flight. Additionally, we address two key methodological questions critical for studying bird flight when using GPS devices in conjunction with path annotation. First, we investigate the sensitivity of the methods by testing how reducing the accuracy of the estimated bird position influences the empirical relationship between movement and wind. Second, we assess how assumed environmental data from two different weather-reanalysis datasets at different spatial resolutions influence what we can determine about the relationships between ground speed (*v*_*g*_), airspeed (*v*_*a*_) and wind conditions.

Modern GPS tags determine position with high accuracy via triangulation using differences in arrival time of satellite borne signals. They also provide instantaneous measures of direction (*d*_*i*_) and speed (*v*_*i*_) based on a Doppler-shift information that a moving tag relative to the movement of the satellites causes. The precision of these *d*_*i*_ and *v*_*i*_ measurements is unclear, and before the current study it was uncertain whether they conveyed any ecologically relevant information in addition to flight speed and direction determined using standard methods from sequential GPS locations. In addition to using *v*_*i*_ and *d*_*i*_, we therefore also calculated speed and direction from the next location (*v*_*nl*_ and *d*_*nl*_, respectively). Finally, in order to investigate the potential additive effects of a higher amount of error in determining the true location of the animals, we included a dataset where we added one moderate random shift to each GPS location. The results from this dataset can inform us about the importance of measurement accuracy, which can degrade when using other methods of bird tracking, for example telemetry from radio tags or from the ARGOS satellites. In this dataset, the new positions were picked at random from a 2-dimensional Gaussian distribution containing 95% of the cumulative probability in a circle of 2 km radius around the true GPS-determined location, corresponding to the level of accuracy obtained in the three highest quality levels of ARGOS satellite telemetry (e.g. [34]). For these new positions we calculated direction (*d*_*nl2k*_) and speed (*v*_*nl2k*_) to the next (also randomly shifted) location and correspondingly interpolated the wind speed and direction to these new locations.

**Figure 1:**
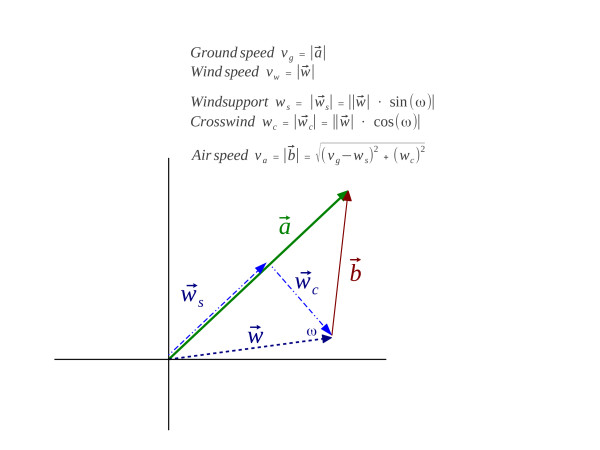
**Schematic representation of the calculated measures, where α represents the vector of a bird's movement relative to the ground.** Its length is *v*_*g*_. Wind support (*w*_*s*_) is the length of the wind vector in the direction of c and cross-wind (*w*_*c*_) the length of the perpendicular component. Finally, airspeed (*v*_*a*_) is the speed of the bird relative to the wind and can be calculated as given above, or modeled as the intercept of a model with *v*_*g*_as a function of *w*_*s*_and *w*_*c*_ *.*

The Movebank Track Annotation Tool was used to annotate the wind direction and speed for each location using a weighted distance interpolation of two different data sources: 1) The ERA-interim data provided by the European Centre for Midrange Weather Forecast (ECMWF; http://ww.ecmwf.int/products/data/archive/descriptions/ei/index.html) [35] and 2) The National Centers for Environmental Prediction (NCEP) and Atmospheric Research (NCAR) Global reanalysis-II dataset, (http://www.esrl.noaa.gov/psd/data/gridded/data.ncep.reanalysis2.html) [36, 37]. Using this information, our overall goal was to model *v*_*g*_ and *v*_*a*_ as a function of wind support (*w*_*s*_) and cross wind (*w*_*c*_). Wind support (*w*_*s*_) was calculated as the length of the wind vector in the direction of the birds' flight where positive values represent tail wind and negative values head wind. Cross wind (*w*_*c*_) represented the speed of the wind vector perpendicular to the travel direction irrespective of which side it came from (Figure 1).

Using the two different weather models and the different ways of obtaining the birds' flight direction and ground speed, we quantified the amount of total variance explained (as measured by the adj. *R*^*2*^) by the empirical model that predicted ground speed (*v*_*g*_) as a function of *w*_*s*_, *w*_*c*_, and their interaction term. The model intercept represents airspeed (*v*_*a*_) under no wind condition as it is modeled as the speed of the birds when *w*_*s*_ and *w*_*c*_ are zero and was compared to the geometrically calculated values of *v*_*a*_ according to the equation given in Figure 1.

Thus, we provide an assessment of the effects of wind on avian flight across a variety of species and, at the same time, provide a qualitative assessment of the significance of earlier findings derived with location information of lower quality. This will facilitate the comparison of results across studies, which used different methods to track birds and infer wind conditions.

## Results

**Figure 2:**
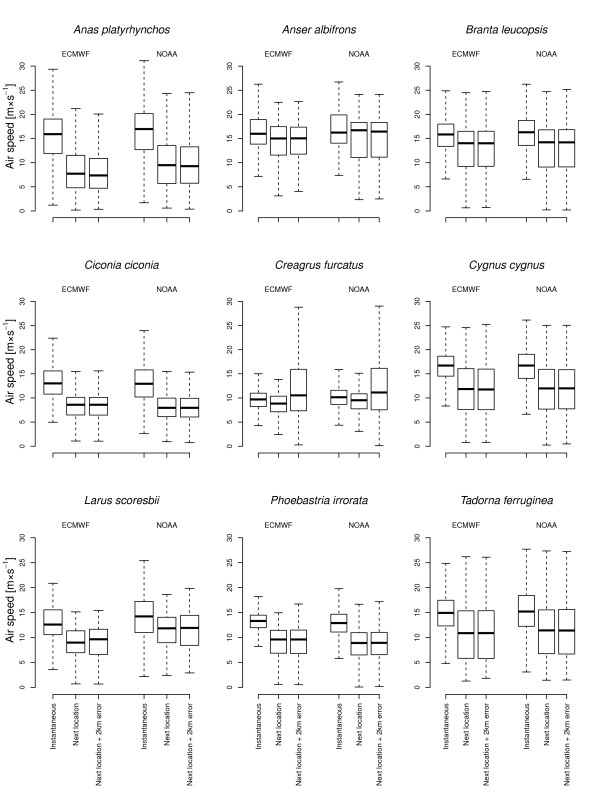
**Box plots of airspeed in meters per second calculated as given in Figure** 5 **using different methods of determining flight direction and ground speed.** The dark line is the median, the box represents the lower and upper quartile and the whiskers are the 1.5 inter-quartile distance. Outliers were omitted.

Despite their different spatial resolution, the two wind datasets resulted in the same overall outcome, with negligible differences between the model estimates (Figure 2 and additional files 1, 2, 3, 4). In general, the analysis based on the ECMWF dataset resulted in higher adj. *R*^*2*^ than the NCEP reanalysis-II dataset (mean decrease in adj. *R*^*2*^±SD=0.07±0.06)*.* In fact, *w*_*s*_ and *w*_*c*_ values from both models were highly correlated (adj. *R*^*2*^±SD for *w*_*s*_=0.74±0.15 and *w*_*c*_=0.76±0.14). We therefore present the results only for the higher resolution ECMWF wind dataset and provide the calculations based on NCEP reanalysis-II as supplemental on-line material.

Using instantaneous direction (*d*_*i*_) and ground speed (*v*_*i*_) versus direction and ground speed derived from the next location (*d*_*nl*_ and *v*_*nl*_) resulted in very different calculated airspeeds. Airspeed derived from geometric calculation using vector addition was consistently higher for *v*_*i*_ and *d*_*i*_ derived measures (Figure 2 & Table 1).

**Table 1:** Summary of GLMMs run for different species modeling ground speed as a function of wind support (*w*_*s*_), cross wind (*w*_*c*_) and their interaction term (*w*_*s*_ * w_*c*_), using the three methods to determine ground speed and flight direction (instantaneous, next location and next location + 2 km) described in the text.

Species	Median airspeed		***w***_***s***_	***w***_***c***_	***w***_***s***_ * w_***c***_	Adj. ***R*** ^***2***^	Intercept ± SE	N
*Anas platyrhynchos*	15.86	Instantaneous	***	*	NS	0.15	12.63±0.58	1293
		Next location	*	NS	NS	0.02	5.65±0.22	418
		Next location + 2 km Error	*	NS	NS	<0.01	4.61±0.04	1762
*Anser albifrons*	15.98	Instantaneous	***	NS	NS	0.45	14.91±0.6	151
		Next location	NS	NS	NS	0.23	11.43±1.3	78
		Next location + 2 km Error	NS	NS	NS	0.23	11.36±1.29	78
*Branta leucopsis*	15.83	Instantaneous	***	**	*	0.52	14.77±0.23	1124
		Next location	***	NS	NS	0.26	10.66±0.41	661
		Next location + 2 km Error	***	NS	NS	0.26	10.62±0.41	659
*Ciconia ciconia*	13.01	Instantaneous	***	*	NS	0.35	12.98±0.19	1534
		Next location	***	NS	NS	0.22	7.24±0.32	972
		Next location + 2 km Error	***	NS	NS	0.23	7.32±0.25	971
*Creagrus furcatus*	9.69	Instantaneous	***	***	NS	0.49	8.69±0.15	2001
		Next location	***	***	NS	0.30	7.23±0.13	1632
		Next location + 2 km Error	NS	NS	**	<0.01	7.68±0.46	4769
*Cygnus cygnus*	16.71	Instantaneous	***	***	NS	0.56	16.42±0.21	997
		Next location	***	NS	NS	0.16	10.38±0.40	864
		Next location + 2 km Error	***	NS	NS	0.16	10.31±0.40	867
*Larus scoresbii*	12.59	Instantaneous	**	NS	NS	0.31	10.80±0.64	190
		Next location	NS	NS	NS	0.03	5.12±0.36	75
		Next location + 2 km Error	NS	NS	NS	0.03	5.00±0.29	84
*Phoebastria irrorata*	13.30	Instantaneous	***	***	NS	0.66	12.45±0.18	2081
		Next location	*	*	*	0.11	5.90±0.16	1500
		Next location + 2 km Error	*	*	*	0.10	6.02±0.16	1519
*Tadorna ferruginea*	14.94	Instantaneous	***	*	NS	0.10	14.11±0.17	1250
		Next location	NS	NS	NS	<0.01	8.22±-0.45	464
		Next location + 2 km Error	NS	NS	NS	<0.01	8.32±-0.47	462

***= p<0.0001, **= p<0.001, *= p<0.05. N is the number of observations used in the models. SE is standard error of the estimate. Median airspeeds were estimated using instantaneous ground speed measurements and ECMWF data (see also Figure 1 *) with vector addition, whereas the intercept represents an estimate of airspeed using the regressive model under no wind condition (*w_s_ *and* w_c_ *= 0). In both cases a minimum ground speed of 4* ^*m*^ */*_*s*_ *was used to filter locations that could have been stationary animals.*

Across all species, wind support (*w*_*s*_) was significantly and positively correlated with ground speed (*v*_*g*_) as reported by *v*_*i*_ (in nine of nine species *p*<0.05, Table 1). The estimated significance of the effect of cross wind (*w*_*c*_) and the interaction term between *w*_*s*_ and *w*_*c*_ on *v*_*g*_ differed mainly depending on whether instantaneous or next location was used (Table 1), resulting in very different models. Using next location to derive *d*_*nl*_ and *v*_*nl*_ resulted in non-significant contribution of *w*_*s*_ (Table 1) for three species. Using the next location including a 2 km mean error in determining the true location resulted in the loss of the significant contribution of *w*_*s*_ in one more case (Table 1). Without exception, the intercepts of the models representing estimated airspeed under no wind condition, based on the instantaneous values, were higher than with the two other methods (Table 1) and much closer to the calculated median airspeed. Finally, the proportion of the variance explained (adj. *R*^*2*^) was from 1.3 to >100 times higher using instantaneous speed and direction.

**Figure 3:**
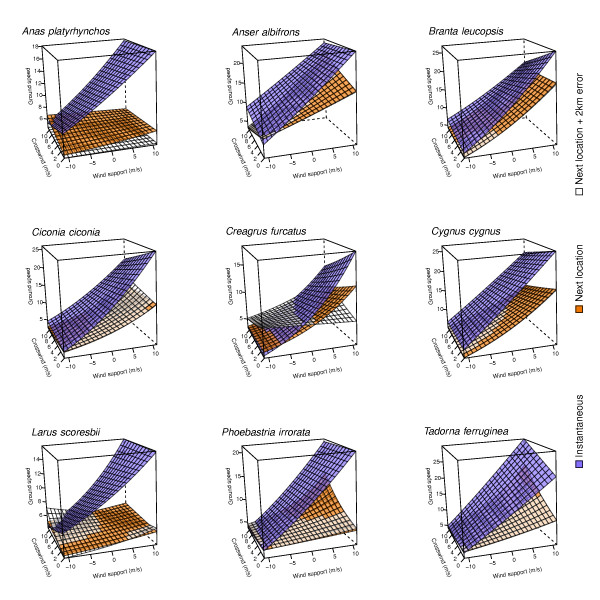
**Predicted ground speed as a function of wind support and cross wind derived from models based on different methods (indicated by color) of determining flight direction and ground speed of 9 different bird species**.

The divergence in *p* values, but particularly the adj. *R*^*2*^ associated with the instantaneous measurements, are indicative of the strength of the effects of *w*_*s*_ and *w*_*c*_ on determining ground speed (*v*_*g*_). At the same time, the decrease in adjusted *R*^*2*^ with measurements using next location with or without the addition of an error indicate that the true effects of wind support and cross winds on determining *v*_*g*_ (and *v*_*a*_ for that matter) are lost with decreasing resolution (Figure 3 and Table 1). The estimated model parameters also diverged in their predictions of the shape of the relationship between *w*_*s*_ and *w*_*c*_ on *v*_*g*_ (Figure 3). Whereas the predicted values from the models based on instantaneous values resulted in a somewhat consistent appearance across all species, the models based on next location with or without error (purple, white and orange grids respectively, Figure 3) were less congruent in how they predicted wind support and cross winds to influence ground speed. In general, *v*_*g*_ decreased with increasing *w*_*c*_, and if a significant interaction was present, this effect became stronger with increasing *w*_*s*_ (with the exception of *Tadorna ferruginea* which showed the opposite pattern).

**Figure 4:**
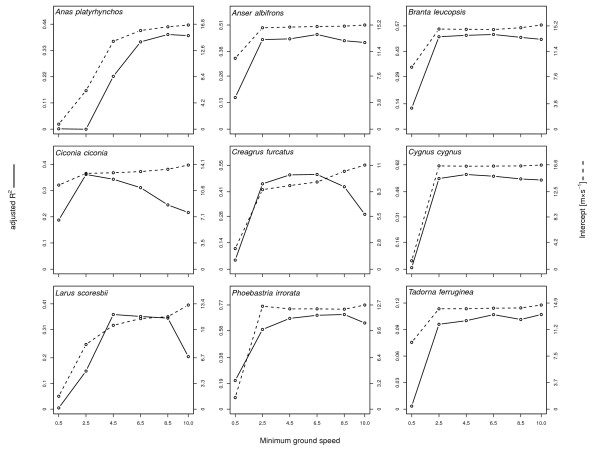
**Estimated proportion of explained variance (adj.** ***R*** ^***2***^ **: solid line) and intercept (dashed line) as a function of minimum ground speed (** ***v***_***g***_ **) starting at 0.5** ^**m**^ **/**_**s**_ **stopping at 10** ^**m**^ **/**_**s**_ **.** Ground speed was modeled using generalized linear mixed models predicted by instantaneous direction (*d*_*i*_) and speed (*v*_*i*_) with individual as random effect and including a temporal autoregressive function to account for spatio-temporal autocorrelation.

Choosing different minimum speed thresholds for filtering the data prior to analysis revealed that, in most species, the proportion of explained deviance (adj. *R*^*2*^) and the estimated intercept already become stable at minimum speeds as low as ≥2^m^/_s_ and remained so at higher speeds. This suggests that the selection of *v*_*i*_ ≥ 4^m^/_s_ excluded stationary locations efficiently. The only exception might be the mallard (*Anas platyrhynchos*) where raising the minimum ground speed was accompanied by a substantially higher estimated intercept. If minimum ground speed for the mallard was raised by only 1^m^/_s_ to a minimum speed of 5^m^/_s_ the intercept was 15.1^m^/_s_ which is probably a more accurate estimate of the intercept and closer to the calculated median airspeed. Using a large minimum speed resulted in a drop in adjusted *R*^*2*^ for some species (notably *Ciconia ciconia*, *Creagrus furcatus* and *Larus scoresbii*) due to a reduction in their sample sizes (Figure 4).

**Figure 5:**
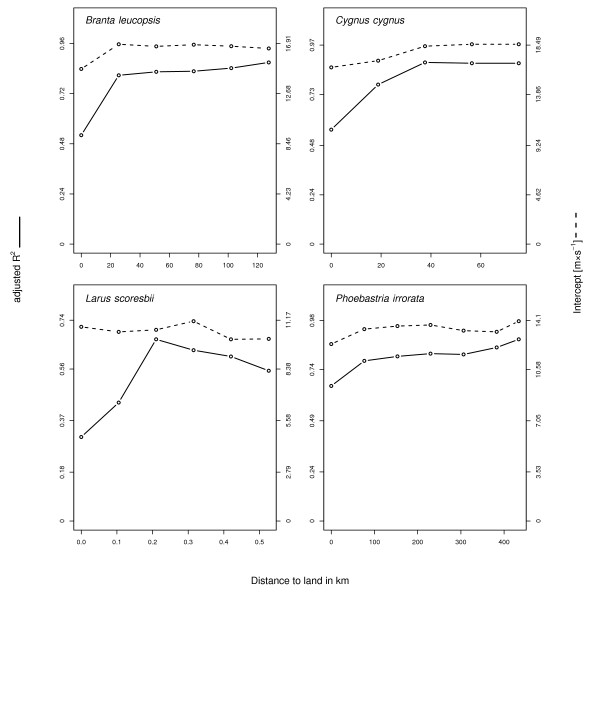
**Adjusted** ***R*** ^***2***^ **(solid line) and model intercept (dashed line) as a function of distance to land using generalized linear mixed models predicting ground speed dependent on wind support, cross wind and their interaction term with individual as random effect and accounting for temporal autocorrelation.** Since the distances at which birds were observed from land differs, the axis of distance to land have different ranges.

Finally, for four species, we compared how well models predicted ground speed as a function of wind support, cross wind and their interaction term at different distances to the land (Figure 5). With increasing distance to land the proportion of the variance explained increased markedly compared to including data from flights both overland and over the sea.

## Discussion

Satellite tracking and remote sensing data now allow us to ask fundamental biological questions about large-scale phenomena such as bird migration [17, 38]. However, as with any new technology, the new data types must be evaluated and compared to traditional methods. Not surprisingly, we found that traditional estimates of flight speed (from sequential, low-resolution locations) resulted in underestimates of the true distance traveled and translated into lower estimates of ground speed [39]. Even in those species where we had a position every 15 or 30 minutes (e.g. some individuals of *Anas platyrhynchos* or *Larus scoresbii*), the differences between using instantaneous measures and next-location measurements were substantial and accompanied by a significant drop in the amount of explained variance. This indicates that deriving speed and direction from the next location is strongly influenced by scaling-dependent measurement biases, as has also been found for measurements of movement paths of terrestrial animals [39–41]. We found that the GPS-based instantaneous direction (*d*_*i*_) and speed (*v*_*i*_) were highly informative and recommend researchers use these parameters to address questions about the movement ecology of tracked animals. Indeed, our results show that even simple models using global weather models can explain an hitherto unachieved 66% of the variance in the observed ground speed (Table 1).

Clearly, using instantaneous measures of movement in combination with weather observations-model hybrid reanalyzes explained a substantially higher proportion of the original deviance than models using consecutive locations. The use of next location, more precisely the time resolution of sampling, in combination with the specific tortuosity of the trajectories, inevitably results in an underestimation of ground speed. This suggests that past studies based on GPS or satellite tracking were likely to have underestimated the influence of wind support and cross winds on the ground speed, and for that matter airspeed of birds. Studies that estimate flight speed using radar [15], double theodolite systems [42], or other immediate observational techniques are less prone to underestimate bird speed, but are limited by where and when birds can be measured. This result is also evident from the stark differences in the patterns (Figure 3) which are a consequence of the combined differences in estimated intercept and parameter estimates of wind support and cross wind. Finally, comparing our estimates of airspeed with published direct observational data derived from radar observations for example published in [16] are strikingly similar (within one standard deviation of the published mean airspeeds) yet consistently lower. These differences might be the consequence of the bias in the location and times observation was possibly with radar, or attributable to an insufficient resolution of weather models.

The use of consecutive location as opposed to instantaneous measures is not only less accurate, but also fundamentally alters the modeled relationships with environmental variables, however, with the exception of the swallow-tailed gull, the noise added to the location data had a negligible effect. Adding a 2 km error to each location adds proportionally much more noise to the flight directions and speed of highly sampled tracks of slow-moving birds, such as the swallow-tailed gulls, than to trajectories with lower sampling frequency of fast flying species. The relative influence of measurement error is thus proportional to the speed of the animals, which might explain the exception we found for the effect of adding a 2 km error. The faster individuals move, the smaller this influence of measurement error will become on the angular velocity determining the noise in measuring flight direction and on misjudgment of the distance and thus speed. Therefore, the measurement error of current tags is likely to define a lower limit of reliably determining speed and direction.

Although instantaneous flight direction and speed are not available for all tag types, they could be estimated by collecting short bursts of GPS location at high resolution (*e.g.* 1*Hz*). Often, the limiting factor in acquisition of locations using GPS tags is battery capacity, however, once the GPS unit has established connection to the satellites and “knows” its location, obtaining additional high frequency locations comes at comparatively low energetic costs. It will be interesting to compare the accuracy of measuring flight directions and speed obtained by high frequency GPS acquisition compared to the Doppler-shift based instantaneous measures of direction and speed in the light of the different measurement accuracies.

Airspeed can be calculated by vector addition or estimated statistically as the model intercept under conditions without winds. Whereas determining mean airspeed from the vector addition is susceptible to selection of minimum *v*_*i*_ by shifting the distribution of speeds towards higher values, and also changes due to the behavioral response to the wind conditions, the statistical model intercepts were very stable across a wide range of minimum selected speeds. Such stable measures of airspeed could be used as more reliable measures for comparative studies for example, or to test hypothesis of airspeed change under specific migratory stages [9, 14, 15].

While most studies using direct methods of measuring ground speed to derive airspeed show a bias towards reporting maximum airspeed as opposed to averages or distributions, the model intercept represents an average over long spatial and temporal periods for a very particular condition of no wind influence. Airspeed is predicted to show a non-linear relationship with wind support and cross wind [1, 22, 24, 28], a fact that was not further investigated in this study. In fact, we removed this non-linearity by transforming the data, as our prime interest was to model ground speed as a function of different wind models and ways of calculating speed and direction. Theory, supported by empirical evidence, predicts that with increasing head wind (negative wind support values) the birds should increase their airspeed [1, 21, 23, 27]. The same is true, in general, for cross wind, where more cross wind should be compensated by increasing airspeed. The fact that the median calculated airspeed tended to be higher than the model intercept might in fact be a consequence of this non-linear relationship. On average, in most species in our data-set, either cross winds had large positive values or wind support was negative, suggesting that most birds in fact experienced head winds and/or cross wind in our samples. So the differences in estimated intercept and the calculated airspeed could be interpreted as a consequence of the fact that the birds rarely flew under no-wind conditions but rather under conditions that required increased airspeed.

Although the choice of the wind dataset did not result in fundamental differences in our study, the resolution of the wind models might still play an important role as the partially substantial increase in adj. *R*^*2*^ showed for increasing distance to land. Global weather models are probably less accurate over land than over open sea due to land-cover and topographic heterogeneity making predictions of local wind conditions very challenging [18]. At the same time the nominal accuracy in determining GPS location and/or the instantaneous measures of movement might be higher over open sea than land. In the two species that did actually fly at substantial distances from land, i.e. larger than at least one single grid-cell size of the weather models (roughly 167 km at the equator), the increase in adj. *R*^*2*^ was 35% (*Branta leucopsis*) and 23% (*Phoebastria irrorata*), reaching a maximum adj. *R*^*2*^ of 0.87 and 0.89 respectively, between the complete data set and the subset containing only the locations farthest from land. Assuming that the accuracy of predicting local wind conditions generally limits the predictive power of the empirical models, actual bird flight speeds are likely to be even more strongly affected by wind conditions than anticipated. It would thus be interesting to investigate the change in performance of high resolution regional atmospheric models in predicting the relationship of flight speed and wind conditions compared to the coarse grain information we used here.

## Conclusions

The remote methods used to measure speed and direction strongly influenced the derived estimates of wind influences on bird flight. Our results suggest that high spatio-temporal resolution of movement data is essential in determining the true effects of wind on ground speed on a micro-scale and thereby determines our estimates of airspeed. When addressing ecological questions involving the relative benefit of wind conditions, additional knowledge or assumptions concerning directional preferences and/or scales of movement may be required. Future studies investigating bird flight in relation to environmental conditions where the speed and flight direction are important entities have to account for scaling effects (*e.g.*[41, 43]). This knowledge will help to improve future tracking studies and advocates for high-resolution temporal (and spatial) data. However, even despite the coarse resolution, the access to global atmospheric wind models already proves an important step towards a better understanding of avian flight in the wild.

## Methods

Data were collected from GPS tags supplied by two manufacturers (Microwave Telemetry Inc., Columbia, MD, USA and E-obs GmbH, Gruenwald, Germany), deployed on 288 individuals from nine species, which yielded a total of 333,082 location records along their tracks (Table 2). The usual measurement error in GPS tags of these manufacturers is on average around ±3m with maximum error below ±20 m over land. The tracks represent both migration and breeding range behavior. The tags, using the latest GPS technology, acquire the position according to a user defined (and thus tag-specific) schedule (Table 2). In addition to location coordinates and timestamps, the GPS units provide a so-called “instantaneous heading” (*d*_*i*_) and “instantaneous speed” (*v*_*i*_) with every acquisition of a position. Although the term used in reporting the measurement is originally “heading”, it represents actually the birds' flight direction. As the term “heading” in movement analysis very often refers to the direction of the body axis, we refer to the “instantaneous heading” as flight direction (*d*). The instantaneous direction (*d*_*i*_) and speed (*v*_*i*_) are derived from the Doppler-shift that a moving tag produces in relation to the movement of the GPS satellites, they are thus actual observations of “instantaneous” flight direction and speed at the time of acquisition at a given location. Doppler-shift determination of direction and speed requires, however, a substantial minimal speed of the tag (and bird that carries it), because the accuracy of the detection of the amplitude of the change in frequency of the Doppler-shift decreases at low velocities (and in very high velocities, but these are not relevant to the typical range of migration flight speeds).

**Table 2:** Species composition and sample size used in the study

Species	Latin name	Individuals	Locations	Time range	Schedule (min between fixes)
Mallard	*Anas platyrhynchos*	108	149543	Nov. 2008 – Nov. 2010	15 and 90
White-fronted goose	*Anser albifrons*	4	3382	Feb. 2008 – Sep. 2008	120
Barnacle goose	*Branta leucopsis*	27	35448	Apr. 2006 – Sept. 2009	120
White stork	*Ciconia ciconia*	4	9685	Mar. 2009 – May 2010	60
Swallow-tailed gull	*Creagrus furcatus*	16	9249	Aug. 2008 – Jul. 2009	5
Whooper swan	*Cygnus cygnus*	56	59315	Aug. 2007 – Sep.2009	60
Dolphin gull	*Larus scoresbii*	18	2847	Jan. 2009	30
Waved albatross	*Phoebastria irrorata*	29	16140	May – Nov. 2008	90
Ruddy shelduck	*Tadorna ferruginea*	26	47473	Mar. 2007 – Mar. 2010	120

Studies investigating the interaction between ground speed (Figure 1: *v*_*g*_), airspeed (Figure 1: *v*_*a*_) and wind have so far derived direction (*d*_*nl*_), ground speed (*v*_*g*_) and airspeed (*v*_*a*_) from differencing two or more points along the sequences of locations, which, depending on the schedule of the tags, are based on various time intervals. We used great distances between the subsequent locations in conjunction with the time differences to calculate speed from the next locations.

For each position, the shortest great circle distance between the bird and the nearest land was determined using the Global Self-consistent, Hierarchical, High-resolution Shoreline database (GSHHS) (http://www.ngdc.noaa.gov/mgg/shorelines/gshhs.html). The Movebank Track Annotation Tool annotated the wind direction and speed for each location, using a weighted distance interpolation. The ECMWF ERA-interim weather model has a global resolution of 1.5°, which corresponds to 167 km at the equator, and the NCEP data resolves at 2.5° at a global scale corresponding to 278 km at the equator. For the interpolation of wind we used surface-level winds interpolated to 10m above ground, as most birds flew within the depth of the lowest resolved atmospheric pressure layer in the weather models. Vertical interpolation to the GPS-recorded height did not alter the performance of our statistical tests on comparison with surface level wind speed and directions (data not shown).

For each species, method of calculating flight direction and for each weather dataset, a generalized linear mixed model (GLMM) was used. Temporal autocorrelation was accounted for by including a continuous autoregressive process for a continuous time-covariate. Because most data sets had enough data per individual, we limited the minimum number of observations to 12 per individual, except for *Larus scoresbii* where there were few samples per individual so data for all individuals were combined. The identity of individual birds (individual ID) was included as a random factor in the GLMM to account for individual differences in airspeed. We excluded all locations with ground speeds <4^m^/_s_ (indicating stationary animal or discontinuous movement) and speeds >30^m^/_s_ (as there was a high probability that these outliers were measurement errors). Thus, we derived predictive models of ground speed as a function of wind support and cross winds and their interaction accounting for the individual variance and the spatio-temporal autocorrelation present in the data.

Theoretical and empirical evidence suggests that birds should increase airspeed with increasing head wind and cross winds (relative to intended travel directions) causing a non-linear effect of wind support and cross wind on airspeed. The consequence of this non-linearity is that it violates the assumptions of linear models by introducing heteroscedasticity and a deviation of the residuals from normality. Therefore, before running the GLMM, we transformed the data using a Box-Cox transformation and used a Gaussian error distribution. Due to the Box-Cox transformation, however the model estimates of the slopes cannot be cross-compared directly. For an intuitive model comparison, we therefore plot the model predictions for ground speed over the range of observed wind support and cross winds (Figure 2). We assessed the proportion of total variance explained in the models by using adjusted *R*^*2*^ (adj. *R*^*2*^), defined as the proportion of the residual variance over original variance, both estimated using the unbiased estimators [44]*.*

In addition, we quantified the extent to which sensor limitations that lead to a required minimum speed threshold influences the model fit (adj. *R*^*2*^) and estimate of the intercept (estimate of *v*_*a*_) when using *d*_*i*_ and *v*_*i*_. For this analysis, we followed the same procedure as described above but for a range of minimum thresholds starting at 0.5^m^/_s_ to 10^m^/_s_ in steps of 2^m^/_s_. First, we selected a minimum speed, filtered out all observations with instantaneous speed lower than that threshold, transformed the remaining (above threshold) data using a Box-Cox transformation and ran a GLMM as described above. Finally, to assess the effect of distance to land for those individuals that flew over sea, we quantified model fit and intercept in relation to the distance to land using the same procedure with a minimum-speed threshold for *v*_*i*_ of 4^m^/_s_. For this analysis the data for each analysis was split into 20 subsets using 20 minimum distances to land spanning the observed range of distances to land equally.

Observations and experiments on *Anas platyrhynchos, Ciconia ciconia, Cygnus cygnus* were conducted under German permits and according to German law licenced to the Max Planck Institute for Ornithology. Experiments on *Creagrus furcatus* were conducted as part of a tri-party agreement between the Max Planck Institute for Ornithology, the Charles-Darwin Research Station and the Galapagos National Park Service and permitted by Ecuadorean law. Tagging and tracking methods for *Phoebastria irrorata* was reviewed and approved by the Galapagos National Park Service. *Larus scroesbi* were studied under permissions from the Falkland New Island Conservation Trust. Tracking of *Cygnus cygnus* and *Branta leucopsis* was undertaken with the approval of Wildfowl and Wetlands Trust's Animal Welfare and Ethics Committee and under license from the British Trust for Ornithology. Procedures for capture, handling, and marking of *Anser albifrons* and *Tadorna ferruginea* were approved by a U.S. Geological Survey Animal Care and Use Committee and the University of Maryland Baltimore County Institutional ACUC (Protocol EE070200710). Ethical approval for trapping, sampling, and keeping of the Swedish *Anasplatyrhynchos* was obtained from the Swedish Animal Research Ethics Board (“Linköpings djurförsöksetiska nämnd”, reference number 61-10).

## Electronic supplementary material

Additional file 1: **Predicted ground speed as a function of wind support and cross wind derived from models based on different methods (indicated by color) of determining flight direction and ground speed of 9 different bird species based on the lower resolution global weather model of the National Centers for Environmental Prediction (NCEP) and Atmospheric Research (NCAR).** (PDF 114 KB)

Additional file 2: **Estimated proportion of explained variance (adj.** ***R*** ^***2***^ **: solid line) and intercept (dashed line) as a function of minimum ground speed (** ***v***_***g***_ **) starting at 0.5** ^**m**^ **/**_**s**_ **stopping at 10** ^**m**^ **/**_**s**_ **based on the lower resolution global weather model of the National Centers for Environmental Prediction (NCEP) and Atmospheric Research (NCAR).** Ground speed was modeled using generalized linear mixed models predicted by instantaneous direction (*d*_*i*_) and speed (*v*_*i*_) with individual as random effect and including a temporal autoregressive function to account for spatio-temporal autocorrelation. (PDF 9 KB)

Additional file 3: **Adjusted** ***R*** ^***2***^ **(solid line) and model intercept (dashed line) as a function of distance to land using generalized linear mixed models predicting ground speed dependent on wind support, cross wind and their interaction term with individual as random effect and accounting for temporal autocorrelation.** The analysis is based on the lower resolution global weather model of the National Centers for Environmental Prediction (NCEP) and Atmospheric Research (NCAR). Since the distances at which birds were observed from land differs, the axis of distance to land have different ranges. (PDF 7 KB)

Additional file 4: **Summary of GLMMs run for different species modeling ground speed as a function of wind support (** ***w***_***s***_ **), cross wind (** ***w***_***c***_ **) and their interaction term (** ***w***_***s***_ * w_***c***_ **), using the three methods to determine ground speed and flight direction (instantaneous, next location and next location + 2 km) described in the text.** ***= p<0.0001, **= p<0.001, *= p<0.05. N is the number of observations used in the models. SE is standard error of the estimate*.* Median airspeeds were estimated using instantaneous ground speed measurements and NCAR/NCEP data with vector addition, whereas the intercept represents an estimate of airspeed using the regressive model under no wind condition (*w*_*s*_and *w*_*c*_= 0). In both cases a minimum ground speed of 4^m^/_s_ was used to filter locations that could have been stationary animals. (DOC 44 KB)
