# Supplementary material for: Flying with the wind: scale dependency of speed and direction measurements in modelling wind support in avian flight
Source: Mov Ecol. 2013 Jul 3;1(1):4. doi: 10.1186/2051-3933-1-4 (PMC4337751; doi:10.1186/2051-3933-1-4)
Supplement: Supplementary file 1 — Additional file 1: Predicted ground speed as a function of wind support and cross wind derived from models based on different methods (indicated by color) of determining flight direction and ground speed of 9 different bird species based on the lower resolution global weather model of the National Centers for Environmental Prediction (NCEP) and Atmospheric Research (NCAR). (PDF 114 KB) [file 40462_2013_4_MOESM1_ESM.pdf]

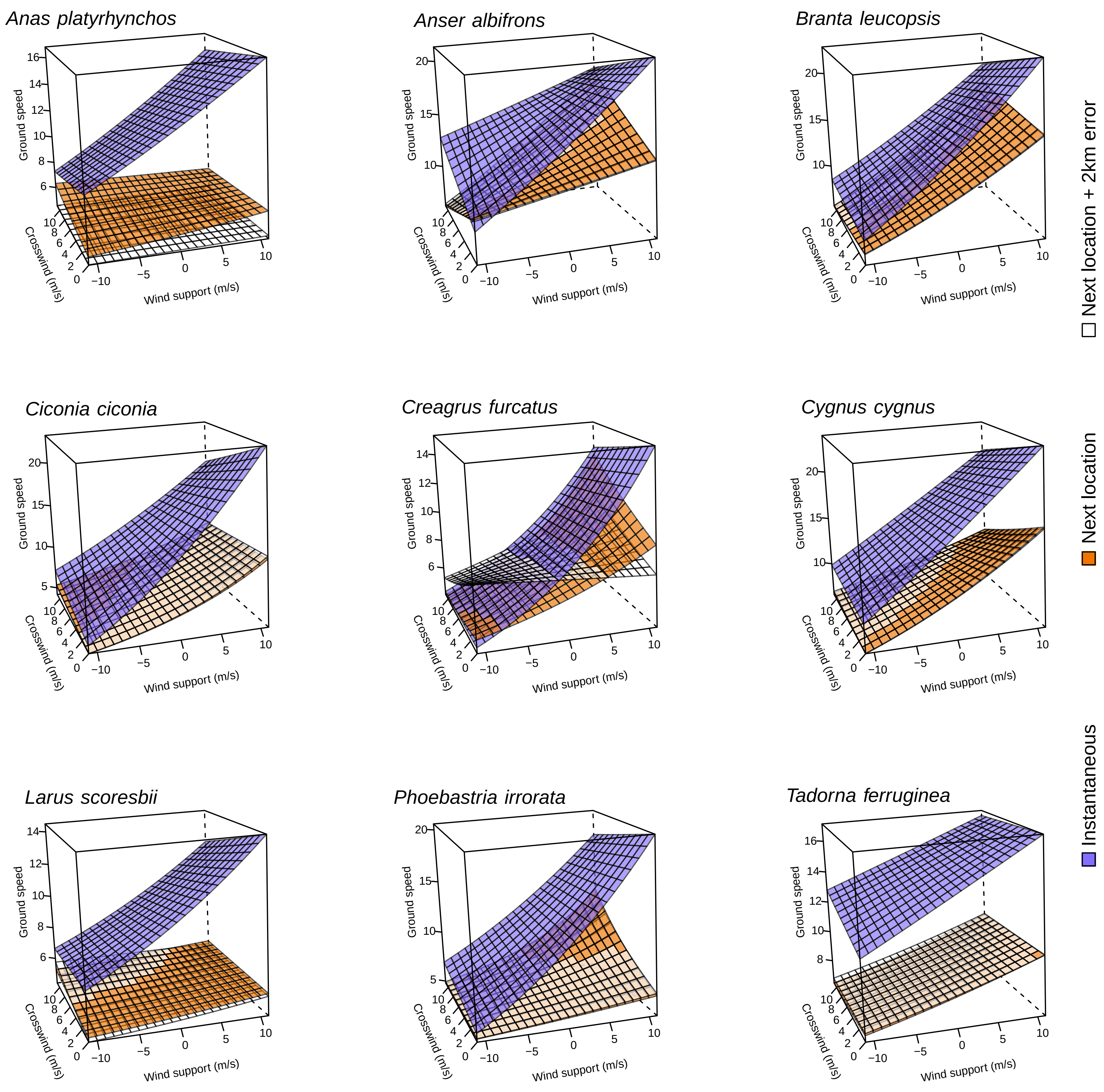

Anas platyrhynchos
Anser albifrons
Branta leucopsis
Ciconia ciconia
Creagrus furcatus
Cygnus cygnus
Larus scoresbii
Phoebastria irrorata
Tadorna ferruginea
Ground speed
Crosswind (m/s)
Wind support (m/s)
Next location + 2km error
Next location
Instantaneous
