# Supplementary figures and images for: Flying with the wind: scale dependency of speed and direction measurements in modelling wind support in avian flight
Source: Mov Ecol. 2013 Jul 3;1(1):4. doi: 10.1186/2051-3933-1-4 (PMC4337751; doi:10.1186/2051-3933-1-4)

adjusted  $R^2$  —

Intercept [ $\text{m}\cdot\text{s}^{-1}$ ] - -

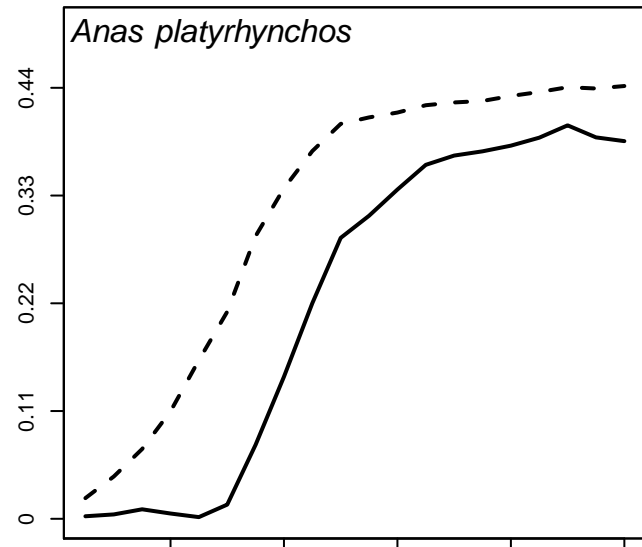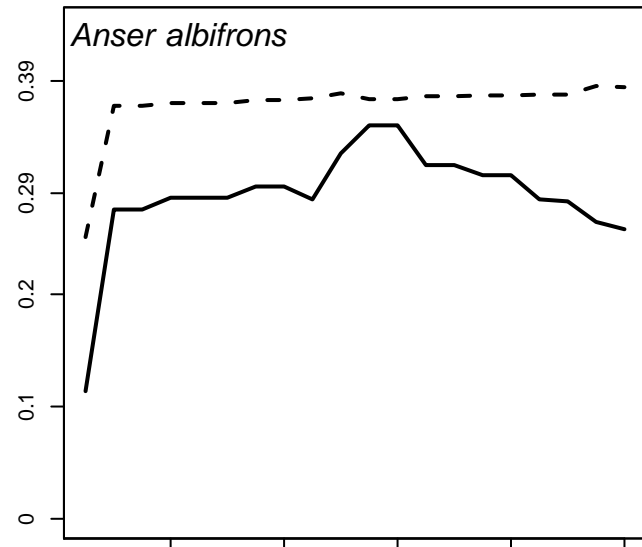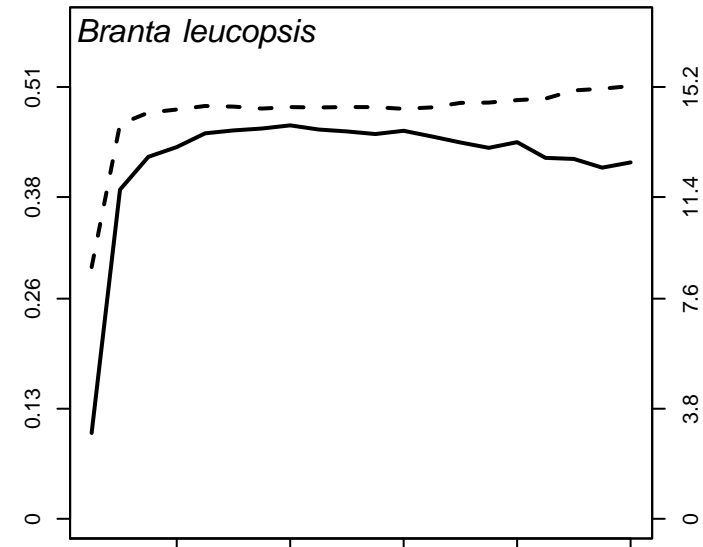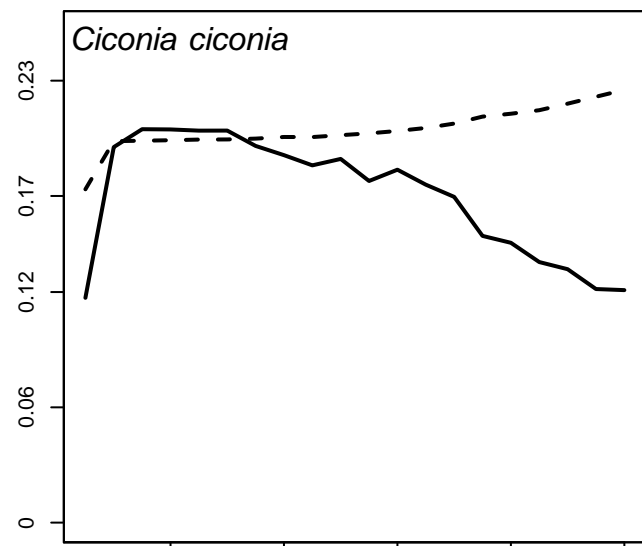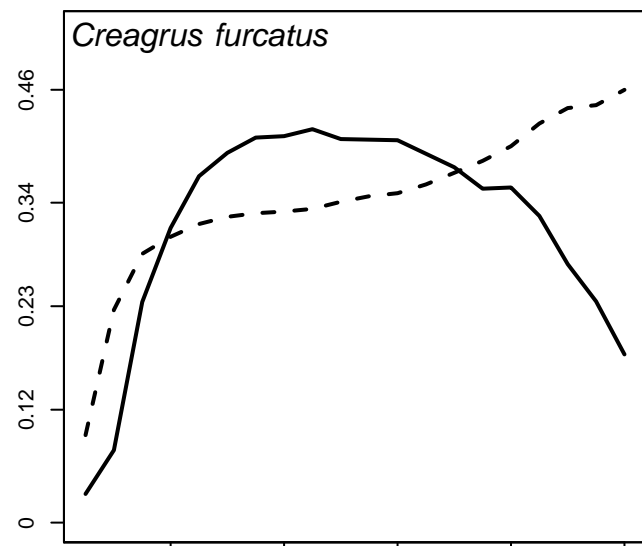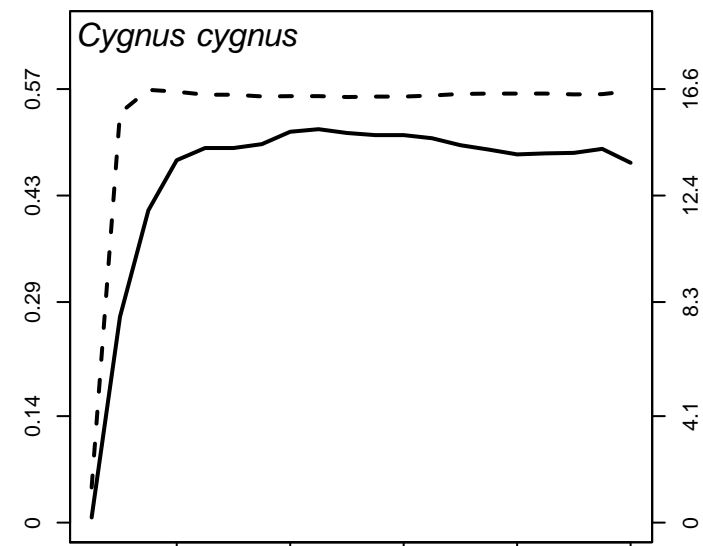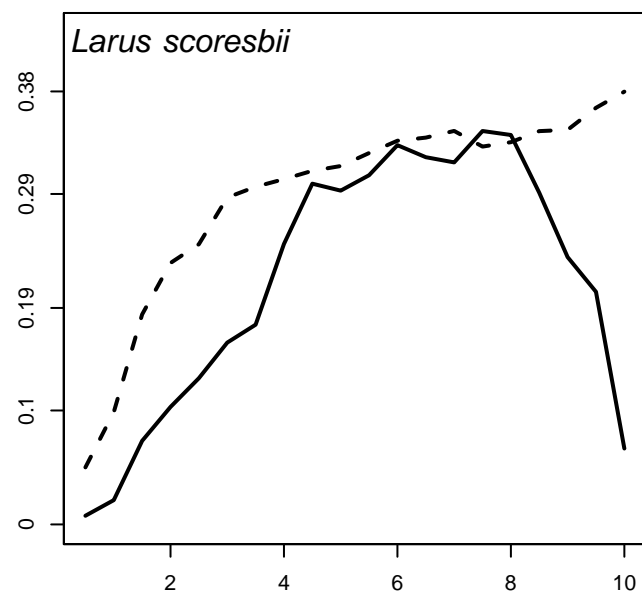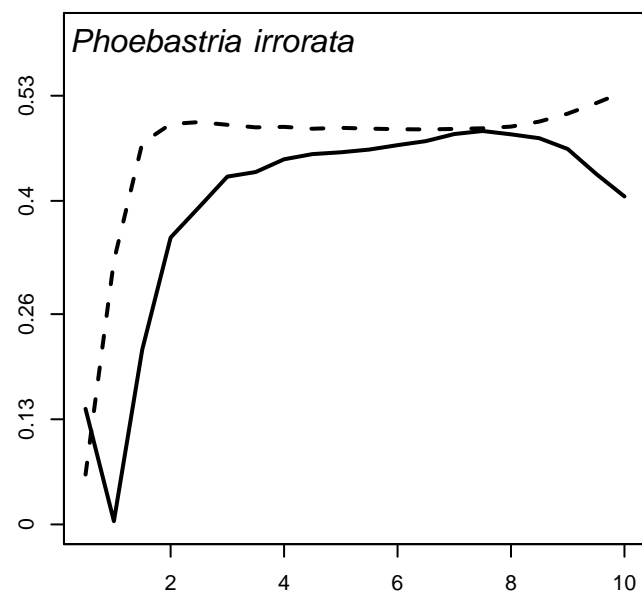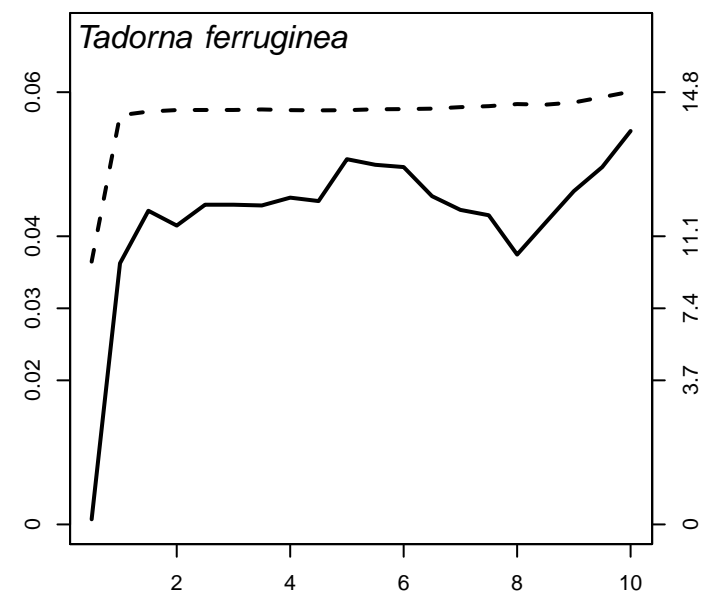

Minimum ground speed

Supplement: Supplementary file 2 — Additional file 2: Estimated proportion of explained variance (adj. R 2 : solid line) and intercept (dashed line) as a function of minimum ground speed ( v g ) starting at 0.5 m / s stopping at 10 m / s based on the lower resolution global weather model of the National Centers for Environmental Prediction (NCEP) and Atmospheric Research (NCAR). Ground speed was modeled using generalized linear mixed models predicted by instantaneous direction (d i) and speed (v i) with individual as random effect and including a temporal autoregressive function to account for spatio-temporal autocorrelation. (PDF 9 KB) [file 40462_2013_4_MOESM2_ESM.pdf]

adjusted R<sup>2</sup> —

Intercept [mxs<sup>-1</sup>] - - -

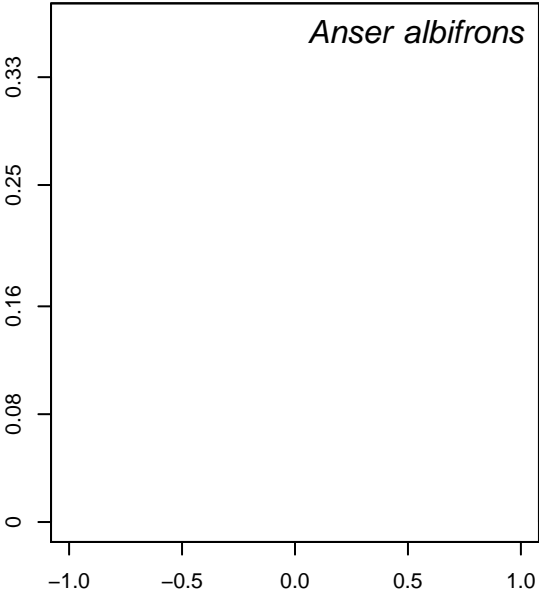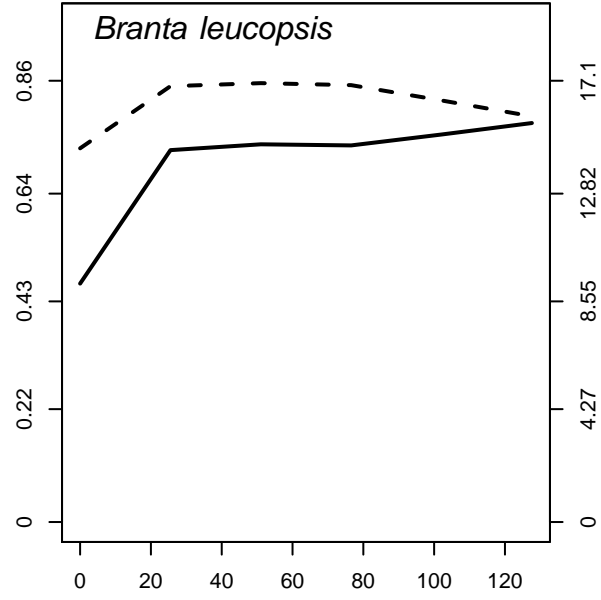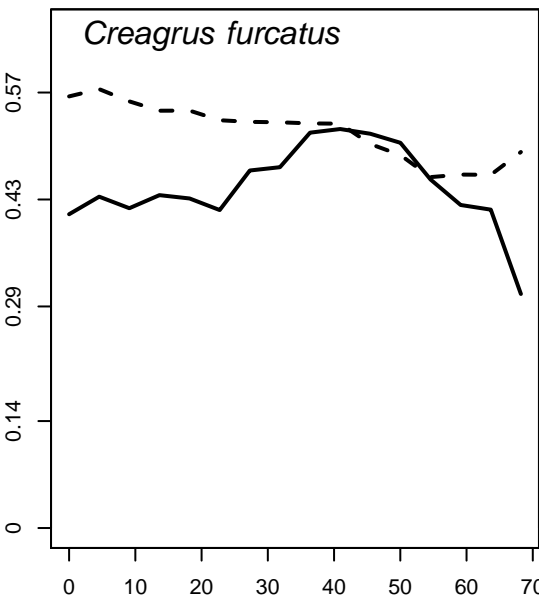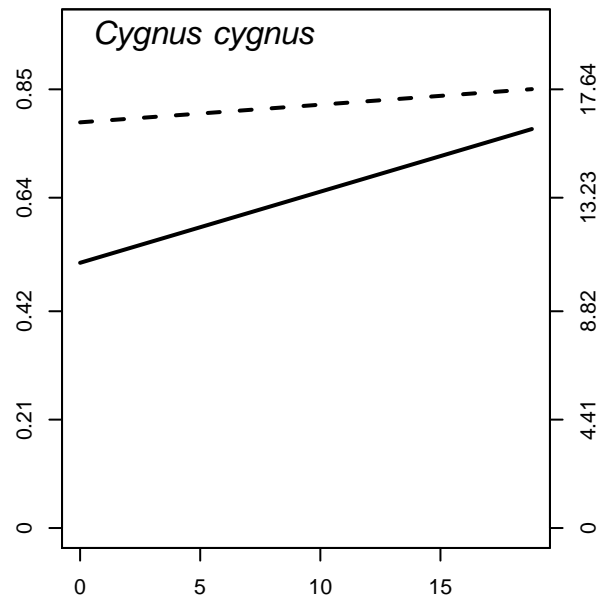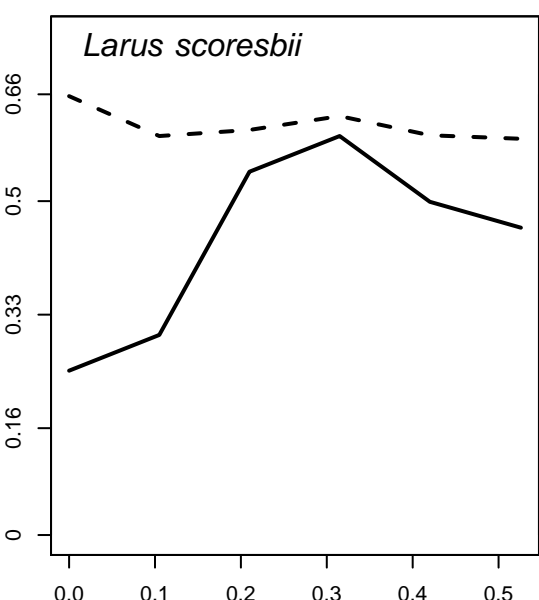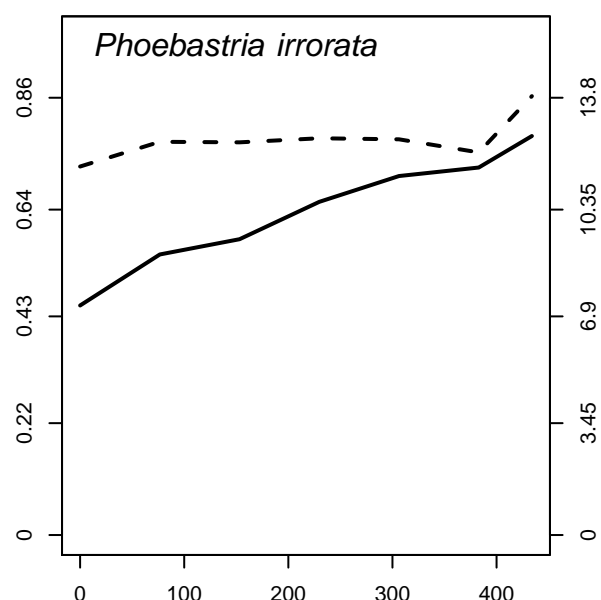

Distance to land in km

Supplement: Supplementary file 3 — Additional file 3: Adjusted R 2 (solid line) and model intercept (dashed line) as a function of distance to land using generalized linear mixed models predicting ground speed dependent on wind support, cross wind and their interaction term with individual as random effect and accounting for temporal autocorrelation. The analysis is based on the lower resolution global weather model of the National Centers for Environmental Prediction (NCEP) and Atmospheric Research (NCAR). Since the distances at which birds were observed from land differs, the axis of distance to land have different ranges. (PDF 7 KB) [file 40462_2013_4_MOESM3_ESM.pdf]
