# Supplementary material for: Flying with the wind: scale dependency of speed and direction measurements in modelling wind support in avian flight
Source: Mov Ecol. 2013 Jul 3;1(1):4. doi: 10.1186/2051-3933-1-4 (PMC4337751; doi:10.1186/2051-3933-1-4)
Supplement: Supplementary file 4 — Additional file 4: Summary of GLMMs run for different species modeling ground speed as a function of wind support ( w s ), cross wind ( w c ) and their interaction term ( w s * w c ), using the three methods to determine ground speed and flight direction (instantaneous, next location and next location + 2 km) described in the text. ***= p<0.0001, **= p<0.001, *= p<0.05. N is the number of observations used in the models. SE is standard error of the estimate. Median airspeeds were estimated using instantaneous ground speed measurements and NCAR/NCEP data with vector addition, whereas the intercept represents an estimate of airspeed using the regressive model under no wind condition (w sand w c= 0). In both cases a minimum ground speed of 4m/s was used to filter locations that could have been stationary animals. (DOC 44 KB) [file 40462_2013_4_MOESM4_ESM.doc]

*Summary of the GLMM output based on the global wind model provided by the National Centers for Environmental Prediction (NCEP) and Atmospheric Research (NCAR) Global reanalysis-II dataset. *** indicate p<0.0001, ** indicate p<0.001, * indicate p<0.05. Wind support is abbreviated as w_s_ and cross wind as w_c_, the interaction term is indicated by w_s_ * w_c_. N is the number of observations used in the models. SE is standard error of the estimate.*

| Species | Median air speed |  | w_s_ | w_c_ | w_s_ * w_c_ | Adj. R^2^ | Intercept ± SE | N |
| --- | --- | --- | --- | --- | --- | --- | --- | --- |
| *Anas platyrhynchos* | 11.70 | Instantaneous | *** | *** | NS | 0.14 | 12.75±0.58 | 1293 |
|  |  | Next location | ** | NS | * | 0.02 | 5.69±0.22 | 418 |
|  |  | Next location + 2 km Error | * | * | NS | <0.01 | 4.63±0.04 | 1762 |
| *Anser albifrons* | 16.43 | Instantaneous | *** | NS | NS | 0.30 | 14.70±0.6 | 151 |
|  |  | Next location | NS | NS | NS | 0.17 | 11.34±1.4 | 78 |
|  |  | Next location + 2 km Error | NS | NS | NS | 0.17 | 11.24±1.4 | 78 |
| *Branta leucopsis* | 15.29 | Instantaneous | *** | * | * | 0.46 | 14.46±0.23 | 1124 |
|  |  | Next location | *** | NS | NS | 0.23 | 10.56±0.42 | 661 |
|  |  | Next location + 2 km Error | *** | NS | NS | 0.22 | 10.48±0.41 | 659 |
| *Ciconia ciconia* | 9.76 | Instantaneous | *** | NS | NS | 0.19 | 12.53±0.24 | 1534 |
|  |  | Next location | *** | NS | NS | 0.11 | 6.78±0.36 | 972 |
|  |  | Next location + 2 km Error | *** | NS | NS | 0.12 | 6.86±0.30 | 971 |
| *Creagrus furcatus* | 10.27 | Instantaneous | *** | * | NS | 0.41 | 8.27±0.18 | 2001 |
|  |  | Next location | *** | NS | * | 0.22 | 6.62±0.13 | 1632 |
|  |  | Next location + 2 km Error | NS | NS | * | 0.01 | 7.83±0.47 | 4769 |
| *Cygnus cygnus* | 14.18 | Instantaneous | *** | ** | * | 0.51 | 16.32±0.22 | 997 |
|  |  | Next location | *** | * | NS | 0.14 | 10.66±0.42 | 864 |
|  |  | Next location + 2 km Error | *** | * | NS | 0.14 | 10.58±0.42 | 867 |
| *Larus scoresbii* | 12.98 | Instantaneous | *** | NS | NS | 0.25 | 10.29±0.62 | 190 |
|  |  | Next location | NS | NS | NS | 0.01 | 5.10±0.41 | 75 |
|  |  | Next location + 2 km Error | NS | NS | NS | 0.05 | 4.86±0.29 | 84 |
| *Phoebastria irrorata* | 10.67 | Instantaneous | *** | NS | * | 0.45 | 11.63±0.14 | 2081 |
|  |  | Next location | * | *** | * | 0.12 | 5.76±0.14 | 1500 |
|  |  | Next location + 2 km Error | * | *** | NS | 0.11 | 6.86±0.14 | 1519 |
| *Tadorna ferruginea* | 13.92 | Instantaneous | *** | NS | NS | 0.05 | 14.14±0.17 | 1250 |
|  |  | Next location | NS | NS | NS | <0.01 | 8.33±-0.47 | 464 |
|  |  | Next location + 2 km Error | NS | NS | NS | <0.01 | 8.40±-0.48 | 462 |
